# lncRNA HCG11 Promotes Colorectal Cancer Cell Malignant Behaviors via Sponging miR-26b-5p

**DOI:** 10.1155/2023/9011232

**Published:** 2023-02-23

**Authors:** Song Guo, Bingtan Song, Lin Li, Hesheng Li, Tao Yang, Lianmeng Cao, Jian Wang

**Affiliations:** ^1^Department of Gastrointestinal Surgery, Binzhou Medical University Hospital, No. 661 Huanghe 2nd Road, Binzhou City, 256603 Shandong Province, China; ^2^Operating Room, Binzhou Medical University Hospital, Binzhou City, 256603 Shandong Province, China; ^3^Department of Gastroenterology & Endoscopy, Binzhou Medical University Hospital, Binzhou City, 256603 Shandong Province, China

## Abstract

Colorectal cancer (CRC) is a type of gastrointestinal cancer with an increasing incidence. Long noncoding RNAs (lncRNAs) have raised great concern because of wide participation in human diseases, including cancers. However, whether lncRNA HLA complex group 11 (HCG11) played a functional role in CRC remained to be elucidated. Herein, we utilized qRT-PCR to analyze the expression of HCG11 and found that HCG11 was highly expressed in CRC cells. Besides, HCG11 knockdown suppressed cell proliferation, migration, and invasion but facilitated cell apoptosis. Furthermore, supported by bioinformatics analyses and mechanism assays, HCG11, mainly located in cell cytoplasm, was confirmed to competitively bind to miR-26b-5p to modulate the expression of the target messenger RNA (mRNA), namely, cAMP-regulated phosphoprotein 19 (ARPP19). ARPP19 was detected to be upregulated in CRC cells, and ARPP19 silence was verified to inhibit the malignant behaviors of CRC cells. Rescue experiments validated that miR-26b-5p inhibition or ARPP19 overexpression could countervail the inhibitory influences of HCG11 silence on CRC cell biological behaviors in vitro. To conclude, HCG11, upregulated in CRC cells, could promote cell proliferation, migration, and invasion and inhibit cell apoptosis via targeting miR-26b-5p/ARPP19 axis.

## 1. Introduction

Colorectal cancer (CRC) considered as a most prevalent cancer in the digestive system is a critical cause of mortality, severely impacting health and life quality of people worldwide [1]. CRC mortality rate declined during the most recent decade (2010-2019) by roughly 2% per year, but this trend masks increasing mortality among young adults. Specifically, CRC death rate rose by 1.2% per year from 2005 to 2019 in individuals younger than 50 and by 0.6% per year in those aged 50-54 [2]. Colonoscopy, characterized with high sensitivity and specificity, has been suggested as the gold standard for CRC detection, but this technique is costly and requires experienced endoscopists and adherence of patients. Endoscopic polypectomy, surgery, radiochemotherapy combinations, and chemotherapeutical regimens mixed with medications are the conventional therapeutic strategies to CRC [3]. Despite impressive gains in molecular genetics of CRC and management of this disease, metastasis is still challenging, and poor treatment outcomes highlight the necessity to obtain a better understanding of molecular mechanisms contributing to CRC initiation and progression [4, 5].

The involvement of long noncoding RNAs (lncRNAs) in modulating cellular processes as well as tumor metastasis and progression has been unveiled in accumulating studies, and some lncRNAs have also been suggested as valuable biomarkers for diagnosis and treatment of multiple malignancies, including CRC [6, 7]. For instance, Lnc-LALC, upregulated in CRC, was proved to negatively regulate LZTS1 expression through affecting DNA methylation, thus accelerating CRC liver metastasis [8]. ABHD11-AS1, displaying a high expression in CRC, was associated with poor prognosis of CRC patients and was validated to exert its oncogenic influences via activating ITGA5/Fak/PI3K/Akt signaling pathway [9]. Additionally, Lnc-S100B-2 was discovered to affect CRC cell apoptosis and the microenvironment via modulation on MLLT10 [10]. lncRNA human leukocyte antigen complex group 11 (HCG11) has been investigated in several malignancies. Specifically, HCG11 was verified to inhibit cell proliferation and cell cycle in osteosarcoma and suppress tumor growth via miR-942-5p/IGF2BP2/p27 Kip1 axis [11]. Moreover, HCG11/miR-26b-5p/QKI5 feedback loop was discovered to reverse vascular endothelial injury induced by high glucose [12]. However, the specific influences of HCG11 in CRC and the relevant molecular mechanism were still elusive.

lncRNAs have been suggested to function as competing endogenous RNAs (ceRNAs) in cancer cells, which compete with protein-coding mRNAs for binding to miRNAs [13, 14]. For example, lnc-PICSAR was corroborated as miR-485-5p sponge to enhance REV3L expression, thus contributing to cisplatin resistance in cutaneous squamous cell carcinoma [15]. Lnc-CTSLP8 was testified as miR-199a-5p sponge to upregulate CTSL1 and thereby exerted oncogenic effects in ovarian cancer [16]. Additionally, lnc-HSD17B11-1:1 was sustained as a ceRNA to promote CRC progression via sponging miR-338-3p and upregulating MACC1 [17]. The function of HCG11 as a ceRNA in CRC cells is still poorly understood.

The main aim in the current study was to investigate the impacts of HCG11 on biological behaviors of CRC cells in vitro and to explore the putative ceRNA network involving HCG11, microRNA-26b-5p (miR-26b-5p), and cAMP-regulated phosphoprotein 19 (ARPP19) in CRC cells.

## 2. Materials and Methods

### 2.1. Ethics Statement

Animal experiments and clinical sample collection were not involved in this study. The implementation of other experiments conducted with cells was approved by Binzhou Medical University Hospital.

### 2.2. Cell Culture

Human CRC cell lines (LOVO, HT-29, and HCT15), normal human colonic epithelial cell line (NCM460), and 293T cells used in this study were available from ATCC (Manassas, VA, USA). DMEM culture medium (C11995500BT, Gibco, Rockville, MD, USA) supplemented with 1% antibiotics (Gibco) and 10% FBS (10270-106, Gibco) was used to culture those cell lines, and all cells were cultured in a humidified incubator (4110; Thermo Fisher Scientific, Shanghai, China) with supplement of 5% CO_2_ at 37°C.

### 2.3. Quantitative Real-Time PCR (qRT-PCR)

qRT-PCR was performed for measurement of RNAs according to published procedures [18]. Total RNA was isolated from cells with application of TRIzol reagent (15596018, Invitrogen, Carlsbad, CA, USA) as per user manual. 500 ng of total RNA was used for cDNA synthesis according to the guidelines of PrimeScript Reverse Transcriptase Kit (2680A, Takara, Shiga, Japan). Then, SYBR Green PCR Kit (RR086A, Takara) was utilized for quantitative analyses. The thermocycling conditions were as follows: 95°C for 30 s, followed by 40 cycles of denaturation at 95°C for 5 s, annealing at 60°C for 30 s, and extension at 72°C for 30 s. GAPDH or U6 was used as the internal reference, and the expression of the detected genes was measured with 2^−*ΔΔ*Ct^ method. The primer sequences used are listed in Table 1.

### 2.4. Cell Transfection

Specific sh-RNAs against HCG11 (sh-HCG11#1/2) or ARPP19 (sh-ARPP19#1/2) and their negative control (sh-NC), along with the pcDNA3.1-ARPP19 and empty vector (pcDNA3.1), were bought from GenePharma (Shanghai, China). Besides, miR-26b-5p mimics/inhibitor or NC mimics/inhibitor (RiboBio, Guangzhou, China) were obtained for miR-26b-5p overexpression or inhibition. Cells were transfected for 48 h in the presence of Lipofectamine 3000 (L3000001, Invitrogen). The sequences of the transfection plasmids are listed in Table 1.

### 2.5. 5-Ethynyl-2′-deoxyuridine (EdU) Assay

EdU assay was conducted as former study has described [19]. BeyoClick™ EdU Cell Proliferation Kit with Alexa Fluor 594 was acquired from Beyotime (C0078S, Shanghai, China) for the implementation of this assay. After 48 h plasmid transfection, cells were incubated with 10 *μ*M EdU medium diluent for 2 h and then counterstained in DAPI solution (D9542, Signal-Aldrich, St. Louis, MO, USA). After being washed in PBS, cells were observed under inverted microscope (NIB410-FL, Mshot). The percentage of EdU-positive cells was counted by dividing the number of EdU-positive cells by that of DAPI-stained cells to reflect changes in cell proliferation.

### 2.6. Colony Formation

As previously described [20], cells after transfection plated into 6-well plates (500 cells in each well) were subjected to 14-day cultivation. Then, the colonies were fixed by formaldehyde (F8775, Merck, Shanghai, China) for 30 min, followed by 5 min staining with 0.5% crystal violet. The visible clones were counted manually.

### 2.7. Terminal Deoxynucleotidyl Transferase (TdT) dUTP Nick-End Labeling (TUNEL) Assay

TUNEL experiments were done for assessment of cell apoptosis as previously described [21]. Transfected cell samples were processed with PBS (SH30256.01B, Hyclone, Logan, UT, USA) and then fixed with 4% paraformaldehyde. 50 *μ*L TUNEL reagent (11373242910, Merck) was applied for staining the apoptotic cells as per the standard protocol, and DAPI was utilized for 10 min counterstaining. The inverted microscopy was used for analyzing, and the percentage of TUNEL-positive cells was calculated.

### 2.8. JC-1 Assay

As previously described [21], the measurement of mitochondrial membrane potential (*Δψ*m) was conducted by JC-1 assay for detection of cell apoptosis. The cancer cells were plated in 12-well plates until cells adhered to the plates and then cultured with 2.5 *μ*g/mL of JC-1 (C2005, Beyotime) at 37°C. After 30 min, samples were observed under inverted microscopy.

### 2.9. Transwell Assay

As previously described [22], cell migratory capacity was measured using 24-well transwell chamber (8 *μ*m; 3410, Costar, Boston, MA, USA), and cell invasive capability was measured with the same chamber coated with Matrigel (356234, BD Biosciences, San Jose, CA, USA). After transfection, cells were collected and resuspended in serum-free culture medium and then added to upper chamber. 100% complete DMEM containing 10% FBS was added to lower chamber as a chemoattractant. After 24 h cell culture, the successfully migrated or invaded cells were stained with crystal violet, and the number of those cells in 5 random fields was counted under inverted microscope.

### 2.10. Fluorescence In Situ Hybridization (FISH)

As previously described [23], cancer cells on slides were first fixed by 4% paraformaldehyde and then processed under a nondenaturing condition, followed by hybridization with HCG11-specific FISH probe (RiboBio). After RNA hybridization, cells were stained with DAPI solution for 5 min and observed under confocal microscopy (DMi8 manual, Leica).

### 2.11. Subcellular Fractionation

As described in the previous study [24], PARIS Kit acquired from Invitrogen (AM1921) was applied for separation of cell cytoplasmic and nuclear fractions. HCG11 content was detected in both cell fractions using qRT-PCR. GAPDH was used as cytoplasmic endogenous control. U6 small nuclear RNA acted as nuclear endogenous control.

### 2.12. RNA Pull-Down

RNA pull-down assay was done as previously described [25]. Biotin-labeled HCG11 probes and control probes were synthesized by RiboBio. Then, the probes were cultured with cell lysates for RNA enrichment. 30 *μ*L of streptavidin magnetic beads (88816) was added into the samples for precipitation. At last, RNAs bound in the precipitated mixture were eluted and analyzed by qRT-PCR.

### 2.13. Luciferase Reporter Assay

As described in recent literature [26], full length of HCG11 or ARPP19 3′UTR fragments covering miR-26b-5p target sites (wild-type and mutant) was acquired and subcloned into pmirGLO dual-luciferase reporter vectors (E1330, Promega, Madison, WI, USA) to construct HCG11-WT/Mut and ARPP19-WT/Mut, which were then cotransfected with miR-26b-5p mimics or NC mimics into 293T cells and cancer cells. After 48 hours, 300 *μ*L of cell lysis buffer was added to lyse the cells. Then, the cell lysates were centrifuged at 13,200 rpm for 2 min to remove cell debris. The relative luciferase intensity (normalized to Renilla luciferase activity) was determined by dual-luciferase reporter assay system (E1910, Promega).

### 2.14. RNA Immunoprecipitation (RIP)

RIP assay was conducted to investigate the RNA potential endogenous interaction using Magna RIP RNA-Binding Protein Immunoprecipitation Kit (17-704, Millipore, Bedford, MA, USA) as previously described [27]. Briefly, cell lysates were cultured with human AGO2 antibody (FNab00214, Fine Test, Wuhan Fine Biotech Co., Ltd.) and control IgG antibody (14678-1-AP, Proteintech, Rosemont, IL, USA) overnight with gentle rotation and then incubated with protein A/G beads (LSKMAGAG, Millipore). After unbound material was washed off, the precipitated RNAs were isolated by resuspending beads in TRIzol RNA extraction reagent and eluted with nuclease-free water. The detection of RNA enrichment was finally completed with qRT-PCR.

### 2.15. Western Blot Assay

RIPA buffer (R0278, Sigma-Aldrich, St. Louis, MO, USA) was utilized to obtain the protein lysates. Then, the concentration of protein was measured by Bradford Protein Assay Kit (orb219873, Biorbyt, Cambridge, England). Later, the proteins were treated with 12% SDS-PAGE (1610174, Bio-Rad Laboratories, Shanghai, China) and transferred to the PVDF membranes (IPVH00010, Millipore). Subsequently, membranes blocked by 5% skimmed milk were incubated with specific primary antibodies including anti-GAPDH (ab188615, Abcam, Cambridge, MA, USA) and anti-ARPP19 (ab8245, Abcam) at 4°C overnight. After that, IgG H&L (HRP) secondary antibody (ab7090, Abcam) was added for 2 h incubation. Eventually, the proteins were measured via enhanced chemiluminescence (ECL) detection system (32134, Pierce Biotechnology, Rockford, IL, USA).

### 2.16. Statistical Analysis

Statistics were analyzed using Prism 6.0 (GraphPad Software, Inc., La Jolla, CA, USA). Data were collected from more than two independent experiments and exhibited as mean ± SD. The significance of differences was valued according to Student's *t*-test or one-way/two-way ANOVA, with *p* value below 0.05 as threshold.

## 3. Results

### 3.1. HCG11 Knockdown Subdues CRC Cell Growth

**Figure 1:**
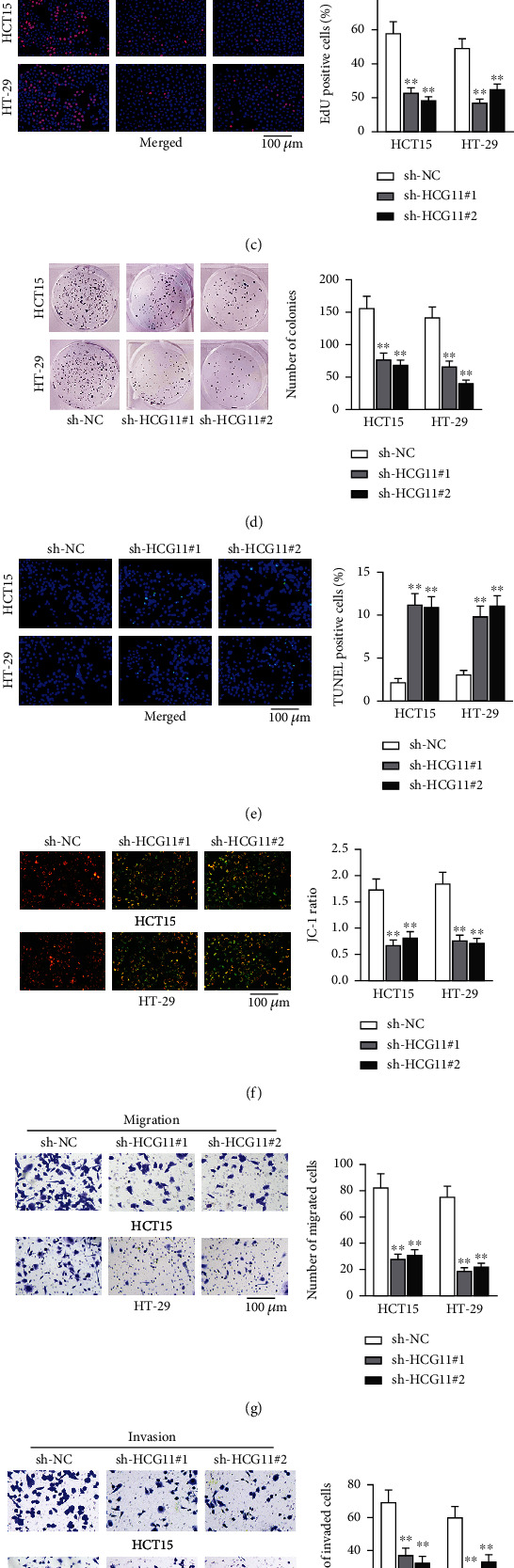
lncRNA HCG11 knockdown subdues CRC cell growth and accelerates cell apoptosis. (a) HCG11 level in CRC cell lines (HCT15, HT-29, and LOVO) and human normal intestinal epithelial cells (NCM-460) was assessed using qRT-PCR. (b) qRT-PCR tested the interference efficiency of sh-HCG11 in HCT15 and HT-29. (c, d) The cell proliferation and colony capacities were evaluated with the assistance of EdU assay and colony formation assay. (e, f) Cell apoptosis was assessed via TUNEL assay and JC-1 assay. (g, h) Cell migration and invasion abilities were tested via transwell assay. ^∗^*p* < 0.05 and ^∗∗^*p* < 0.01.

To ravel out the potential role of HCG11 in CRC, we first predicted the expression of HCG11 in colon adenocarcinoma (COAD) on UALCAN (http://ualcan.path.uab.edu/index.html), and the result noted that HCG11 expression was higher in tumor tissues in comparison with normal tissues (*p* = 1.202370*E* − 02, Figure S1A). Then, we detected HCG11 expression in CRC cell lines and NCM-460 cells with qRT-PCR. As presented in Figure 1(a), HCG11 was aberrantly high in CRC cell lines, including HCT15 (7.718 ± 1.046, *p* < 0.01), HT-29 (7.723 ± 1.001, *p* < 0.01), and LOVO (2.217 ± 0.235, *p* < 0.05) as compared to NCM-460, and the two cell lines with higher HCG11 expression (HCT15 and HT-29) were selected for subsequent experiments. To figure out how HCG11 affected the biological behaviors of CRC cells, we knocked down HCG11 in HCT15 and HT-19 and observed the changes in proliferation and apoptosis. sh-HCG11#1 and sh-HCG11#2 were designed for HCG11 knockdown. Compared with the control group, HCG11 expression was decreased by more than 80% in sh-HCG11#1-transfected cells and by more than 65% in sh-HCG11#2-transfected cells (*p* < 0.01; Figure 1(b)). As depicted in EdU results, the percentage of EdU-positive cells was 58.26% (HCT15) and 49.400% (HT-29) in the sh-NC group, and the percentage decreased to 23.100% (HCT15) and 17.200% (HT-29) as a result of HCG11 silence (*p* < 0.01; Figure 1(c)). In colony formation assay, before HCG11 depletion, the colony numbers of HCT15 and HT-29 cells were, respectively, 157.600 and 142.200, and the colony numbers were almost halved after sh-HCG11#1 transfection (78.260 in HCT15 and 67.300 in HT-29) and sh-HCG11#2 transfection (69.400 in HCT15 and 41.000 in sh-HCG11#2-transfected HT-29) (*p* < 0.01; Figure 1(d)). Moreover, the percentages of TUNEL-positive cells in sh-NC-transfected HCT15 and HT-29 were, respectively, 2.210% and 3.100%, and the percentages increased to 11.230% and 9.890% in sh-HCG11#1-transfected HCT15 and HT-29 and to 10.960% and 11.100% in sh-HCG11#2-transfected HCT15 and HT-29 (*p* < 0.01; Figure 1(e)). JC-1 ratio, originally 1.740 (HCT15) and 1.850 (HT-29) in the control group, was observed to decline as a result of HCG11 knockdown (0.680 and 0.770 in sh-HCG11#1-transfected HCT15 and HT-29 and 0.821 and 0.720 in sh-HCG11#2-transfected HCT15 and HT-29) (*p* < 0.01; Figure 1(f)). The changes in cell migration and invasion were then assessed too. As shown in Figure 1(g), the numbers of migrated cells were 82.800 (HCT15) and 75.400 (HT-29) in sh-NC groups, and after HCG11 silence, the number decreased to 28.000 and 19.000, respectively, in sh-HCG11#1-transfected HCT15 and HT-29 and to 31.300 and 22.000, respectively, in sh-HCG11#2-transfected HCT15 and HT-29 (*p* < 0.01). Furthermore, the numbers of invaded cells were 69.400 (HCT15) and 60.250 (HT-29) in sh-NC groups, and after HCG11 depletion, the numbers were 37.400 (sh-HCG11#1-transfected HCT15), 24.400 (sh-HCG11#1-transfected HT-29), 32.600 (sh-HCG11#2-transfected HCT15), and 33.300 (sh-HCG11#2-transfected HT-29) (*p* < 0.01; Figure 1(h)). All data confirmed that depletion of HCG11 could inhibit the malignant behaviors of CRC cells.

### 3.2. HCG11 Acts as Sponge of miR-26b-5p in CRC Cells

**Figure 2:**
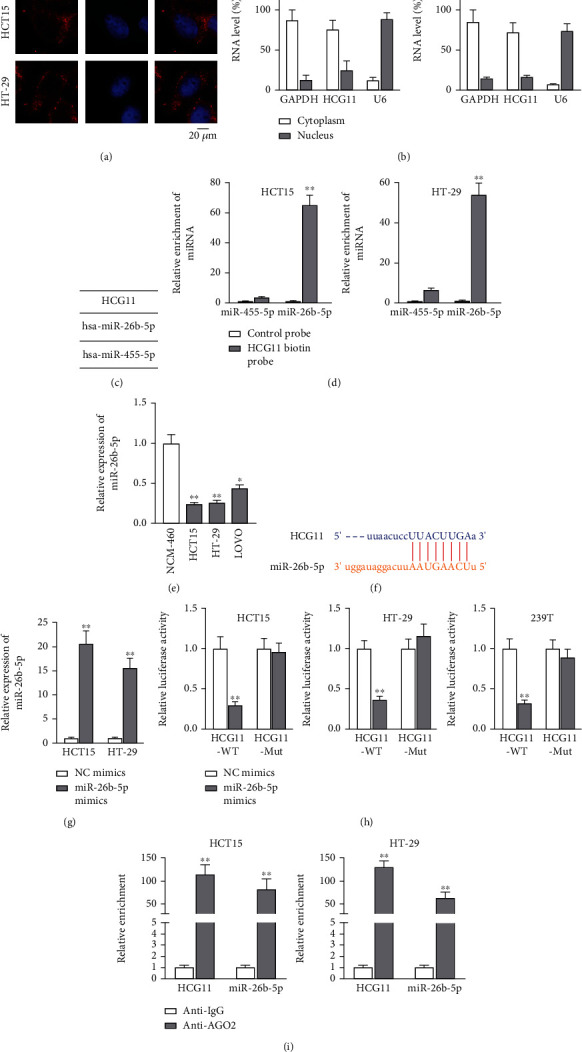
HCG11 sequesters miR-26b-5p in CRC cells. (a, b) Subcellular fractionation and FISH assays were carried out to examine the cellular distribution of HCG11. (c) Putative miRNAs could bind with HCG11 which were predicted based on ENCORI. (d) RNA pull-down assay was conducted to confirm the interplay between HCG11 and miRNAs. (e) miR-26b-5p expression in different cell lines was tested via qRT-PCR. (f) The binding sites of HCG11 and miR-26b-5p obtained from ENCORI were presented. (g) The overexpression efficiency of miR-26b-5p mimics was detected by qRT-PCR. (h) Luciferase reporter assay tested the interplay between HCG11 and miR-26b-5p. (i) RIP assay tested enrichment of HCG11 or miR-26b-5p in anti-AGO2 or anti-IgG complexes. ^∗^*p* < 0.05 and ^∗∗^*p* < 0.01.

To detect the underlying regulatory mechanism of HCG11 in CRC cells, the cellular distribution of HCG11 was firstly testified via FISH assay, and HCG11 was discovered to mainly accumulate in cell cytoplasm (Figure 2(a)). Then, subcellular fractionation experiments were done for further confirmation of the result. The distribution of HCG11 in the cytoplasm of HCT15 and HT-29 cells was, respectively, 75.381% and 72.281%, while that of HCG11 in the nucleus of HCT15 and HT-29 cells was, respectively, 24.618% and 16.376% (Figure 2(b)). Cytoplasmic lncRNAs might function as a ceRNA to bind with miRNAs and modulate the expression of the target genes [13]. Therefore, we also hypothesized that HCG11 could serve as a miRNA sponge in CRC cells. Two miRNAs (miR-26b-5p and miR-455-5p) were predicted with potential to bind with HCG11 on ENCORI (http://starbase.sysu.edu.cn/) database (Figure 2(c)). For further screening, RNA pull-down assay was implemented with control probe and HCG11 biotin probe. In comparison with control probe, the level of miR-455-5p pulled down by HCG11 biotin probe in HCT15 and HT-29 cells was, respectively, 3.612 and 6.574, while the level of miR-26b-5p pulled down by HCG11 biotin probe was, respectively, 65.000 in HCT15 and 54.000 in HT-29 (*p* < 0.01; Figure 2(d)). Thereafter, we conducted qRT-PCR and noticed that miR-26b-5p expressions in CRC cell lines in comparison with NCM-460 cells were 0.24 in HCT15 (*p* < 0.01), 0.26 in HT-29 (*p* < 0.01), and 0.44 in LOVO (*p* < 0.05) (Figure 2(e)). In addition, the binding sites of HCG11 and miR-26b-5p predicted on ENCORI are shown in Figure 2(f). Then, qRT-PCR detected the level of miR-26b-5p after transfection of NC mimics and miR-26b-5p mimics. The results of miR-26b-5p mimic-transfected HCT15 and HT-29 cells were, respectively, 20.666 and 15.666 after being normalized to that in the NC mimic group (*p* < 0.01; Figure 2(g)). As manifested in luciferase reporter assay, the luciferase activity of HCG11-WT was reduced upon miR-26b-5p overexpression relative to the control group (0.290 in HCT15, 0.362 in HT-29, and 0.320 in 293T) while that of HCG11-Mut was barely altered (*p* < 0.01; Figure 2(h)). In RIP assay, results of the anti-AGO2 group normalized to the anti-IgG group were 114.000 (HCG11) and 83.333 (miR-26b-5p) in HCT15 and 130.666 (HCG11) and 64.000 in HT-29 (*p* < 0.01; Figure 2(i)). Taken together, HCG11 acted as sponge to sequester miR-26b-5p in CRC cells.

### 3.3. ARPP19 Is the Downstream Gene of miR-26b-5p

**Figure 3:**
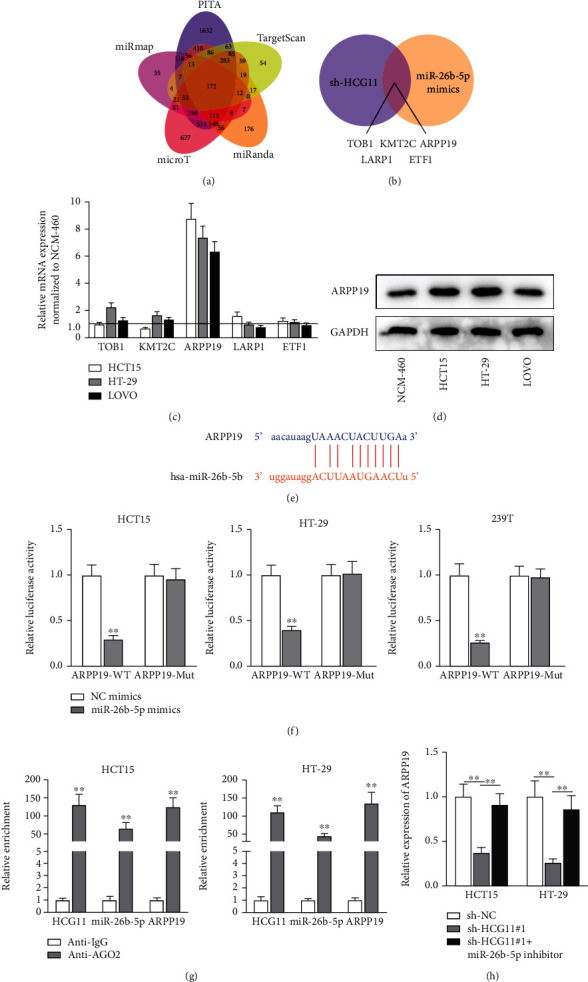
ARPP19 is the downstream gene of miR-26b-5p in CRC cells. (a) The microT, miRanda, miRmap, PITA, and TargetScan databases jointly predicted underlying target genes of miR-26b-5p. (b) The mRNAs downregulated upon HCG11 knockdown and miR-26b-5p overexpression were screened as the candidates. (c) Expressions of 5 candidate mRNAs in CRC cell lines relative to NCM-460 cells were measured via qRT-PCR. (d) Western blot analyzed the expression of ARPP19 in different cell lines. (e) The binding sites between miR-26b-5p and ARPP19 were acquired after ENCORI prediction. (f) Luciferase reporter assay was done to verify the interaction between miR-26b-5p and ARPP19. (g) The existence of HCG11, miR-26b-5p, and ARPP19 in anti-AGO2 was detected by RIP assay. (h) ARPP19 expression was assessed via qRT-PCR in cells with transfection of sh-HCG11#1 or sh-HCG11#1+miR-26b-5p inhibitor. ^∗∗^*p* < 0.01.

To find out the downstream mRNA regulated by miR-26b-5p in CRC cells, microT, miRanda, miRmap, PITA, and TargetScan databases were adopted to screen out the potential target genes (Figure 3(a)). For further screening, qRT-PCR analysis was done to select out candidates modulated by HCG11 and miR-26b-5p, and the expressions of 5 mRNAs (TOB1, KMT2C, ARPP19, LARP1, and ETF1) were detected to be reduced upon sh-HCG11 or miR-26b-5p mimic transfection (Figure 3(b)). The expression levels of the 5 candidates in NCM-460 and CRC cell lines were then measured via qRT-PCR, and only ARPP19 was discovered to be aberrantly upregulated in CRC cell lines (8.782 in HCT15, 7.376 in HT-29, and 6.357 in LOVO) in comparison with NCM-460 (Figure 3(c)). Western blot assay also validated that the protein level of ARPP19 was higher in CRC cell lines in comparison with NCM-460 cells (Figure 3(d)). Additionally, online prediction from UALCAN showed that ARPP19 expression was higher in COAD tissues relative to normal tissues (*p* = 5.683800*E* − 03, Figure S1B). Therefore, ARPP19 was chosen as the research target. The binding sites of ARPP19 and miR-26b-5p predicted on ENCORI are presented in Figure 3(e). Luciferase reporter assay was then done with HCT15, HT-29, and 293T cells, and the luciferase activity of ARPP19-WT was lessened upon miR-26b-5p overexpression (0.296 in HCT15, 0.398 in HT-29, and 0.260 in 293T), while that of ARPP19-Mut remained almost unchanged (*p* < 0.01; Figure 3(f)). In RIP assay, the enrichment of HCG11 (131.333 in HCT15 and 111.000 in HT-29), miR-26b-5p (64.666 in HCT15 and 44.666 in HT-29), and ARPP19 (125.333 in HCT15 and 135.666 in HT-29) in anti-AGO2 was all abundant in comparison with that in anti-IgG (*p* < 0.01; Figure 3(g)). Additionally, qRT-PCR analysis was done to measure ARPP19 expression in cells with inhibition of HCG11 or coinhibition of HCG11 and miR-26b-5p. The expression of ARPP19 reduced to 0.370 in HCT15 and 0.260 in HT-29 as a result of HCG11 knockdown; then, it was recovered to 0.910 and 0.860, respectively, upon cotransfection of miR-26b-5p inhibitor (*p* < 0.01; Figure 3(h)). Taken together, ARPP19 was targeted by HCG11/miR-26b-5p in CRC cells.

### 3.4. ARPP19 Silence Suppresses CRC Cell Growth

**Figure 4:**
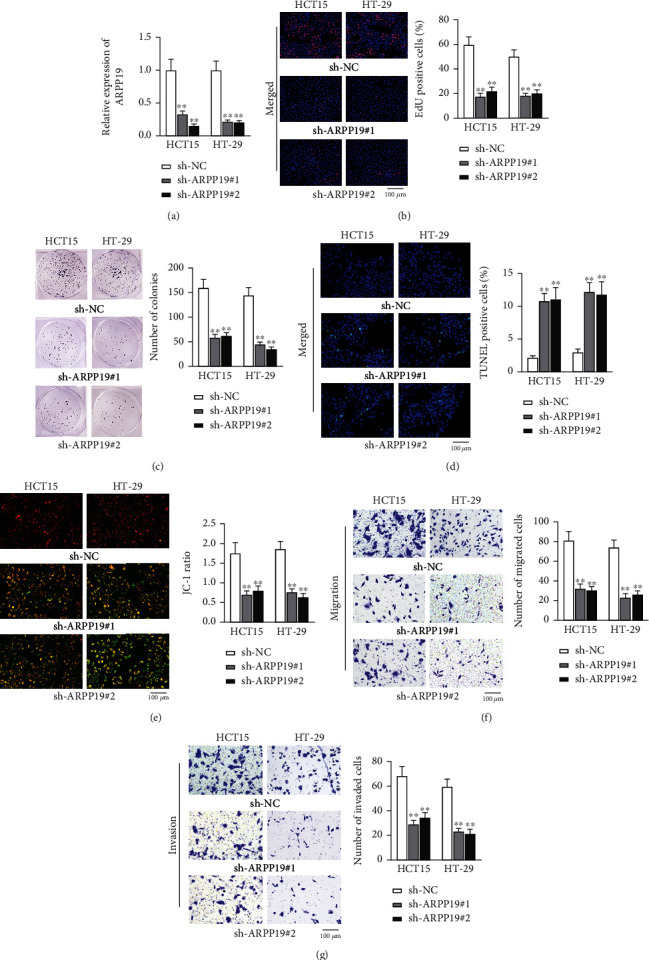
ARPP19 knockdown inhibits CRC cell malignant behaviors. (a) qRT-PCR measured the changes in ARPP19 expression after CRC cells were transfected with sh-NC, sh-ARPP19#1, or sh-ARPP19#2. (b, c) CRC cell proliferation was assessed via EdU assay and colony formation assay. (d, e) The apoptosis capability of transfected cells was evaluated by TUNEL and JC-1 assays. (f, g) Cell migration and invasion abilities were assessed via transwell assay. ^∗∗^*p* < 0.01.

The impacts of ARPP19 on biological behaviors of CRC cells were next investigated. Two plasmids (sh-ARPP19#1/2) were designed for knockdown of ARPP19, and compared with the control group, ARPP19 expression was reduced by roughly 65%-85% after transfection (*p* < 0.01) (Figure 4(a)). As depicted in EdU results, the percentage of EdU-positive HCT15 and HT-29 cells was, respectively, 59.600% and 50.170%, and the percentage decreased to 17.592% and 18.157% in sh-ARPP19#1-transfected groups and to 21.970% and 20.160% in sh-ARPP19#2-transfected groups (*p* < 0.01; Figure 4(b)). In colony formation assay, the number of colonies was, respectively, 159.940 (HCT15) and 144.770 (HT-29), and the colony number decreased to 58.770 (HCT15) and 44.740 (HT-29) after sh-ARPP19#1 transfection and to 62.260 (HCT15) and 35.530 (HT-29) after sh-ARPP19#2 transfection (*p* < 0.01; Figure 4(c)). Moreover, the percentage of TUNEL-positive HCT15 cells increased from 2.170% (sh-NC transfection) to 10.810% (sh-ARPP19#1 transfection) and 11.060% (sh-ARPP19#2 transfection), and that of HT-29 cells increased from 2.990% (sh-NC transfection) to 12.170% (sh-ARPP19#1 transfection) and 11.780% (sh-ARPP19#2 transfection) (*p* < 0.01; Figure 4(d)). In terms of JC-1 ratio, the value in the sh-NC-transfected group was, respectively, 1.750 for HTC15 and 1.861 for HT-29, and ARPP19 knockdown caused a reduction in the value (0.700 and 0.761 in sh-ARPP19#1-transfected HCT15 and HT-29 and 0.800 and 0.640 in sh-ARPP19#2-transfected HCT15 and HT-29) (*p* < 0.01; Figure 4(e)). As shown in Figure 4(f), the number of migrated cells was 81.260 (HCT15) and 74.120 (HT-29) in sh-NC groups, and that was reduced upon ARPP19 silence (32.470 and 23.160 in sh-ARPP19#1-transfected HCT15 and HT-29 and 30.600 and 26.400 in sh-ARPP19#2-transfected HCT15 and HT-29; *p* < 0.01). Furthermore, the number of invaded cells was 68.490 (HCT15) and 59.700 (HT-29) in sh-NC groups and was reduced upon ARPP19 depletion (28.710 and 23.220 in sh-ARPP19#1-transfected HCT15 and HT-29 and 34.500 and 21.330 in sh-ARPP19#2-transfected HCT15 and HT-29) (*p* < 0.01; Figure 4(g)). To sum up, ARPP19 silence weakened the proliferative, migratory, and invasive abilities of CRC cells, while strengthening cell apoptotic ability.

### 3.5. HCG11 Exacerbates CRC Cell Behaviors through Modulating the miR-26b-5p/ARPP19 Axis

**Figure 5:**
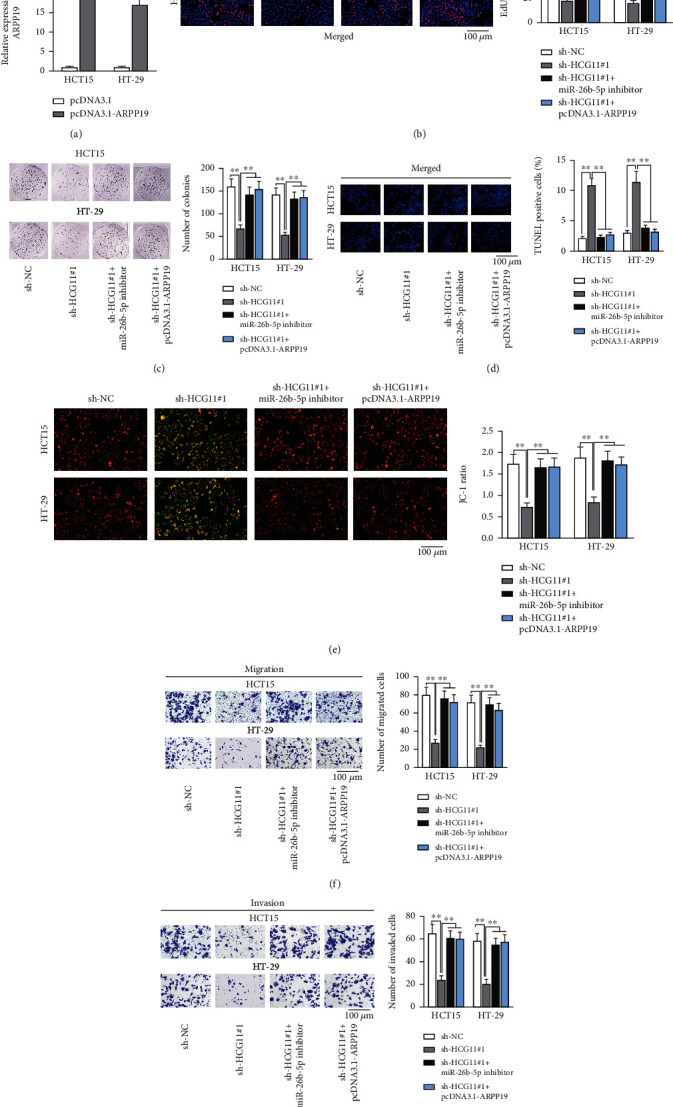
HCG11 exacerbates CRC cell malignant behaviors through modulating the miR-26b-5p/ARPP19 axis. (a) The overexpression efficiency of pcDNA3.1-ARPP19 was assessed through qRT-PCR. HCT15 and HT-29 cells transfected with sh-NC, sh-HCG11#1, sh-HCG11#1+miR-26b-5p, or sh-HCG11#1+pcDNA3.1-ARPP19 were used for functional assays in a rescue manner. (b, c) CRC cell proliferation was evaluated by EdU assay and colony formation assay. (d, e) The cell apoptosis ability was measured via TUNEL assay and JC-1 assay. (f, g) The cell migration and invasion abilities were detected via transwell assay. ^∗∗^*p* < 0.01.

The validity of HCG11/miR-26b-5p/ARPP19 axis in modulation of CRC cell malignant behaviors was next emphasized, and functional assays in a rescue way were designed and carried out with HCT15 and HT-29 cells transfected with indicated plasmids. Firstly, pcDNA3.1-ARPP19 efficiency was determined via qRT-PCR, and compared with the empty vector groups, the level of ARPP19 was augmented to 21.000 in HCT15 and 17.000 in HT-29 (*p* < 0.01; Figure 5(a)). According to the experimental results, the percentage of EdU-positive cells (59.487% in HCT15 and 47.120% in HT-29) reduced upon HCG11 silence (18.126% in HCT15 and 16.167% in HT-29) was almost fully reversed as a result of miR-26b-5p inhibition (57.160% in HCT15 and 42.160% in HT-29) or ARPP19 overexpression (54.160% in HCT15 and 45.267% in HT-29) (*p* < 0.01; Figure 5(b)). Consistently, the number of colonies (159.400 in HCT15 and 141.700 in HT-29) was also noticed to decline upon HCG11 silence (68.300 in HCT15 and 53.500 in HT-29), which was rescued by miR-26b-5p inhibition (142.400 in HCT15 and 133.300 in HT-29) or ARPP19 overexpression (154.500 in HCT15 and 136.600 in HT-29) (*p* < 0.01; Figure 5(c)). In TUNEL assay, the percentage of TUNEL-positive cells (control group; 2.145% and 3.070%, respectively, for HCT15 and HT-29) increased to 10.880% (HTC15) and 11.474% (HT-29) after HCG11 depletion, and the percentage was recovered as a result of miR-26b-5p inhibition (2.400% and 3.890%, respectively, for HCT15 and HT-29) or ARPP19 overexpression (2.760% and 3.240%, respectively, for HCT15 and HT-29) (*p* < 0.01; Figure 5(d)). According to JC-1 staining, miR-26b-5p inhibition or ARPP19 overexpression was detected to countervail the inhibitory influences of HCG11 on JC-1 ratio of HCT15 (sh-NC: 1.733; sh-HCG11#1: 0.727; sh-HCG11#1+miR-26b-5p inhibitor: 1.656; and sh-HCG11#1+pcDNA3.1-ARPP19: 1.677) and HT-29 (sh-NC: 1.883; sh-HCG11#1: 0.844; sh-HCG11#1+miR-26b-5p inhibitor: 1.810; and sh-HCG11#1+pcDNA3.1-ARPP19: 1.711) (*p* < 0.01; Figure 5(e)). The changes in cell migration and invasion were assessed via transwell assays. The decline in number of migrated cells (HCT15: from 80.077 to 27.700 and HT29: from 72.220 to 22.330) induced by HCG11 deficiency was rescued by inhibiting miR-26b-5p (HCT15: 76.530 and HT29: 69.800) or overexpressing ARPP19 (HCT15: 72.200 and HT29: 63.660) (*p* < 0.01; Figure 5(f)). Also, the decrease in invaded cells caused by HCG11 silence (HCT15: from 65.390 to 4.240 and HT29: from 58.440 to 20.780) induced by HCG11 deficiency was rescued as a result of miR-26b-5p inhibition (HCT15: 61.160 and HT29: 55.400) or ARPP19 overexpression (HCT15: 60.330 and HT29: 57.770) (*p* < 0.01; Figure 5(g)). In conclusion, HCG11 exacerbated the malignant behaviors of CRC cells via modulation on miR-26b-5p/ARPP19.

## 4. Discussion

Accumulating researches have unmasked the wide participation of lncRNAs in the regulation of CRC cell biological behaviors and tumor progression [18, 28]. Specifically, AGER-1 exerts inhibitory influences on CRC progression via sponging miR-182 [29] while SNHG10 contributes to exacerbated malignant behaviors of CRC cells via targeting miR-3690 [30]. In this study, we aimed to fathom out the functional role of HCG11 in the regulation of CRC cell malignant behaviors. Based on previous literature, HCG11 was verified as a tumor suppressor in several malignancies, including gastric cancer [31] and cervical cancer [32] while HCG11 was also suggested as an oncogenic factor in ovarian cancer [33]. In the current study, HCG11 was detected to be aberrantly overexpressed in CRC cell lines, and based on results of functional assays, we confirmed that HCG11 silence could promote the apoptosis of CRC cells while inhibiting cell proliferation, migration, and invasion.

The cellular distribution of lncRNAs is suggested to be associated with their specific regulatory mechanisms, and specifically, nuclear lncRNAs might transcriptionally regulate the target genes and cytoplasmic lncRNAs might posttranscriptionally modulate the target genes [23, 34]. Based on FISH and subcellular fractionation results, the main distribution of HCG11 in CRC cell cytoplasm was ascertained, reflecting the putative regulation of HCG11 on downstream gene at posttranscriptional level. CeRNA network is a conventional and crucial posttranscriptional regulatory mechanism involving lncRNAs in CRC [35]. For instance, SNHG8 was sustained as a ceRNA to sponge miR-588 and upregulate ATG7, thus promoting autophagy in CRC [36]. MALAT1 was corroborated as a ceRNA to exert oncogenic influences on CRC [37]. Additionally, HCG11 was verified to sponge miR-942-5p to enhance BRMS1 expression, thus affecting the biological behaviors of gastric cancer cells [31]. Consistent with recent studies, HCG11 was validated as a molecular sponge of miR-26b-5p, targeting ARPP19 to positively modulate ARPP19 expression. Based on previous literature, ARPP19 depletion could suppress the proliferation and migration of CRC cells (SW480) [38]. Moreover, ARPP19 overexpression was corroborated to neutralize the repressive effect of SNHG6 knockdown on the progression of nasopharyngeal carcinoma [39]. Consistently, it was proved via current functional assays that ARPP19 silence could repress the proliferation, migration, and invasion of CRC cells (HCT15 and HT-29), while promoting cell apoptosis. Furthermore, ARPP19 overexpression or miR-26b-5p inhibition was validated to countervail the suppressive influences of HCG11 silence on the malignant behaviors of CRC cells.

In conclusion, the current study has confirmed that lncRNA HCG11, aberrantly upregulated in CRC cells, could exacerbate the malignant behaviors of CRC cells via modulation on miR-26b-5p/ARPP19. The present finding is limited due to lack of animal experiments for further confirmation of the regulatory role of HCG11 in CRC cells in vivo. Moreover, clinical samples need to be collected for validation of the clinical value of HCG11 as a potential biomarker. Nevertheless, a novel molecular perspective might be offered to benefit the understanding of CRC.

## Figures and Tables

**Table 1:** Primer sequences used in qRT-PCR and sequences of transfection plasmids.

Primer sequences used in qPCR	
HCG11	F: TGACTATTCACTGCGTGGTGG
	R: AGGTCACTCCCAGAAGACAG
GAPDH	F: GACAGTCAGCCGCATCTTCT
	R: GCGCCCAATACGACCAAATC
U6	F: TCCCTTCGGGGACATCCG
	R: AATTTTGGACCATTTCTCGATTTGT
miR-455-5p	Reverse transcription stem loop primer: CTCAACTGGTGTCGTGGAGTCGGCAATTCAGTTGAGCcgatgt miRNA stem loop universal reverse primer: CTCAACTGGTGTCGTGGAGTCGGCAATTCAGTTGAGC F: tatgtgcctttggact
miR-26b-5p	Reverse transcription stem loop primer: CTCAACTGGTGTCGTGGAGTCGGCAATTCAGTTGAGCacctat miRNA stem loop universal reverse primer: CTCAACTGGTGTCGTGGAGTCGGCAATTCAGTTGAGC F: ttcaagtaattcagg
TOB1	F: CTAACGGCGATCTCCCAAGG
	R: GTGATCTTGTTCGGGCTGCT
KMT2C	F: GAAGGCAACTGACCCAGGAA
	R: AGACCTGCAGATGTTGCTCC
ARPP19	F: GCGGAGGAGCAGAAGGAAAT
	R: GTTGAGGAAGGTCTTGCGGA
LARP1	F: ATTTACGGCGGCTGCATCTA
	R: GCCTCGAACTTGGCTAGCAG
ETF1	F: GGAGGAGGAGGCGAGAAGAT
	R: GCAGTTCCAAACTCATCCGC

Sequences of transfection plasmids	
sh-NC	5′-CCGGTCATTCTACCATTTGTCTCTTCTCGAGAAGAGACAAATGGTAGAATGATTTTTG-3′
sh-HCG11#1	5′-CACCGTCATTCTACCATTTGTCTCTTCTCGAGAAGAGACAAATGGTAGAATGA-3′
sh-HCG11#2	5′-CACCAATGTCTTCTAAATATGACCACTCGAGTGGTCATATTTAGAAGACA-3′
sh-NC	5′-CCGGTTTATCTTCCATTTCCTTCTGCTCGAGCAGAAGGAAATGGAAGATAAATTTTTG-3′
sh-ARPP19#1	5′-CACCGTTTATCTTCCATTTCCTTCTGCTCGAGCAGAAGGAAATGGAAGATAAA-3′
sh-ARPP19#2	5′-CACCGTTCTTCATTTTTGCTTTAGCCCTCGAGGGCTAAAGCAAAAATGAAGAA-3′
NC mimics	uucaauacaggauaguuguag
miR-26b-5p mimics	uucaaguaauucaggauaggu
NC inhibitor	accuauccuaacuugaagauu
miR-26b-5p inhibitor	accuauccugaauuacuugaa

## Data Availability

The data used to support the findings of this study are included within the article.

## Supplementary Materials

Supplementary Materials Figure S1: (A) HCG11 expression in COAD was predicted on UALCAN. (B) ARPP19 expression in COAD was predicted on UALCAN.

## References

[1] Ma Y. S., Li W., Liu Y., Shi Y., Lin Q. L., Fu D. (2020). Targeting colorectal cancer stem cells as an effective treatment for colorectal cancer. *Technology in Cancer Research & Treatment*.

[2] Siegel R. L., Miller K. D., Fuchs H. E., Jemal A. (2022). Cancer statistics, 2022. *CA: A Cancer Journal for Clinicians*.

[3] Ebrahimzadeh S., Ahangari H., Soleimanian A. (2021). Colorectal cancer treatment using bacteria: focus on molecular mechanisms. *BMC Microbiology*.

[4] La Vecchia S., Sebastián C. (2020). Metabolic pathways regulating colorectal cancer initiation and progression. *Seminars in Cell & Developmental Biology*.

[5] Piawah S., Venook A. P. (2019). Targeted therapy for colorectal cancer metastases: a review of current methods of molecularly targeted therapy and the use of tumor biomarkers in the treatment of metastatic colorectal cancer. *Cancer*.

[6] Oh H. H., Joo Y. E. (2020). Novel biomarkers for the diagnosis and prognosis of colorectal cancer. *Intestinal Research*.

[7] Sun Y., Peng P., He L., Gao X. (2020). Identification of lnc RNAs related to prognosis of patients with colorectal cancer. *Technology in Cancer Research & Treatment*.

[8] Zhang C., Wang L., Jin C. (2021). Long non-coding RNA Lnc-LALC facilitates colorectal cancer liver metastasis via epigenetically silencing LZTS1. *Cell Death & Disease*.

[9] Luo J., Jiang Y., Wu L. (2021). Long non-coding RNA ABHD11-AS1 promotes colorectal cancer progression and invasion through targeting the integrin subunit alpha 5/focal adhesion kinase/phosphoinositide 3 kinase/Akt signaling pathway. *Aging*.

[10] Yi J., Peng F., Zhao J., Gong X. (2022). Prognostic Lnc-S100B-2 affects cell apoptosis and microenvironment of colorectal cancer through MLLT10 signaling. *Journal of Oncology*.

[11] Gu J., Dai B., Shi X. (2021). lncRNA HCG11 suppresses human osteosarcoma growth through upregulating p27 Kip1. *Aging*.

[12] Du J., Han R., Li Y. (2020). LncRNA HCG11/miR-26b-5p/QKI5 feedback loop reversed high glucose-induced proliferation and angiogenesis inhibition of HUVECs. *Journal of Cellular and Molecular Medicine*.

[13] Wu X., Sui Z., Zhang H., Wang Y., Yu Z. (2020). Integrated analysis of lncRNA-mediated ceRNA network in lung adenocarcinoma. *Frontiers in Oncology*.

[14] Braga E. A., Fridman M. V., Moscovtsev A. A., Filippova E. A., Dmitriev A. A., Kushlinskii N. E. (2020). LncRNAs in ovarian cancer progression, metastasis, and main pathways: ceRNA and alternative mechanisms. *International Journal of Molecular Sciences*.

[15] Wang D., Zhou X., Yin J., Zhou Y. (2020). Lnc-PICSAR contributes to cisplatin resistance by miR-485-5p/REV3L axis in cutaneous squamous cell carcinoma. *Open Life Sci*.

[16] Wang X., Li X., Lin F. (2021). The lnc-CTSLP8 upregulates CTSL1 as a competitive endogenous RNA and promotes ovarian cancer metastasis. *Journal of Experimental & Clinical Cancer Research*.

[17] Zhang W., Wang B., Wang Q. (2020). Lnc-HSD17B11-1:1 functions as a competing endogenous RNA to promote colorectal cancer progression by sponging miR-338-3p to upregulate MACC1. *Frontiers in Genetics*.

[18] Wang J., Wang W., Tang Q. (2020). Long non-coding RNA lnc-GNAT1-1 suppresses liver cancer progression via modulation of epithelial-mesenchymal transition. *Frontiers in Genetics*.

[19] Li Y. K., Zhu X. R., Zhan Y., Yuan W. Z., Jin W. L. (2021). NEK7 promotes gastric cancer progression as a cell proliferation regulator. *Cancer Cell International*.

[20] Huang G., Liang M., Liu H. (2020). CircRNA hsa*circRNA*104348 promotes hepatocellular carcinoma progression through modulating miR-187-3p/RTKN2 axis and activating Wnt/*β*-catenin pathway. *Cell Death & Disease*.

[21] Li Y., Jiang A. (2020). ST8SIA6-AS1 promotes hepatocellular carcinoma by absorbing miR-5195-3p to regulate HOXB6. *Cancer Biology & Therapy*.

[22] Qiao Y., Jin T., Guan S. (2021). Long non-coding RNA Lnc-408 promotes invasion and metastasis of breast cancer cell by regulating LIMK1. *Oncogene*.

[23] Cai R., Zhang Q., Wang Y., Yong W., Zhao R., Pang W. (2021). Lnc-ORA interacts with microRNA-532-3p and IGF2BP2 to inhibit skeletal muscle myogenesis. *The Journal of Biological Chemistry*.

[24] Dai X., Li Y., Liu W. (2022). Application of RNA subcellular fraction estimation method to explore RNA localization regulation. *G3 Genes|Genomes|Genetics*.

[25] Kong Z., Wan X., Lu Y. (2020). Circular RNA circFOXO3 promotes prostate cancer progression through sponging miR-29a-3p. *Journal of Cellular and Molecular Medicine*.

[26] Huang Y., Feng G. (2021). MiR-423-5p aggravates lung adenocarcinoma via targeting CADM1. *Thorac Cancer*.

[27] Zheng S., Yang L., Zou Y. (2020). Long non-coding RNA HUMT hypomethylation promotes lymphangiogenesis and metastasis via activating FOXK1 transcription in triple-negative breast cancer. *Journal of Hematology & Oncology*.

[28] Sun W., Nie W., Wang Z., Zhang H., Li Y., Fang X. (2020). Lnc HAGLR promotes colon cancer progression through sponging miR-185-5p and activating CDK4 and CDK6 in vitro and in vivo. *Oncotargets and Therapy*.

[29] Lin M., Li Y., Xian J. (2020). Long non-coding RNA AGER-1 inhibits colorectal cancer progression through sponging miR-182. *The International Journal of Biological Markers*.

[30] Zhang H., Fang Z., Guo Y., Wang D. (2021). Long noncoding RNA SNHG10 promotes colorectal cancer cells malignant progression by targeting miR-3690. *Bioengineered*.

[31] Zhang Q., Yang K., Li J., Chen F., Li Y., Lin Q. (2021). Long noncoding RNA HCG11 acts as a tumor suppressor in gastric cancer by regulating miR-942-5p/BRMS1 axis. *Journal of Oncology*.

[32] Zhang Y., Zhang J., Mao L., Li X. (2020). Long noncoding RNA HCG11 inhibited growth and invasion in cervical cancer by sponging miR-942-5p and targeting GFI1. *Cancer Medicine*.

[33] Li X. F., Hu D. M., Zhao Y. X., Zhang L., Jin Y. (2020). Knockdown of lncRNA HCG11 suppresses cell progression in ovarian cancer by modulating miR-144-3p/PBX3. *European Review for Medical and Pharmacological Sciences*.

[34] Fu S., Wang Y., Li H., Chen L., Liu Q. (2020). Regulatory networks of LncRNA MALAT-1 in cancer. *Cancer Management and Research*.

[35] Guo L., Yang G., Kang Y. (2020). Construction and analysis of a ceRNA network reveals potential prognostic markers in colorectal cancer. *Frontiers in Genetics*.

[36] He C., Fu Y., Chen Y., Li X. (2021). Long non-coding RNA SNHG8 promotes autophagy as a ceRNA to upregulate ATG7 by sponging microRNA-588 in colorectal cancer. *Oncology Letters*.

[37] Zhang C., Yao K., Zhang J., Wang C., Wang C., Qin C. (2020). Long noncoding RNA MALAT1 promotes colorectal cancer progression by acting as a ceRNA of miR-508-5p to regulate RAB14 expression. *BioMed Research International*.

[38] Yu Y., Lai S., Peng X. (2021). Long non-coding RNA MCM3AP-AS1 facilitates colorectal cancer progression by regulating the microRNA-599/ARPP19 axis. *Oncology Letters*.

[39] Yin X., Gu X., Li F., Ye F., Liu F., Wang W. (2021). LncRNA SNHG6 accelerates nasopharyngeal carcinoma progression via modulating miR-26a-5p/ARPP19 axis. *Bioorganic & Medicinal Chemistry Letters*.

