# Supplementary figures and images for: lncRNA HCG11 Promotes Colorectal Cancer Cell Malignant Behaviors via Sponging miR-26b-5p
Source: J Immunol Res. 2023 Feb 23;2023:9011232. doi: 10.1155/2023/9011232 (PMC9981294; doi:10.1155/2023/9011232)

A

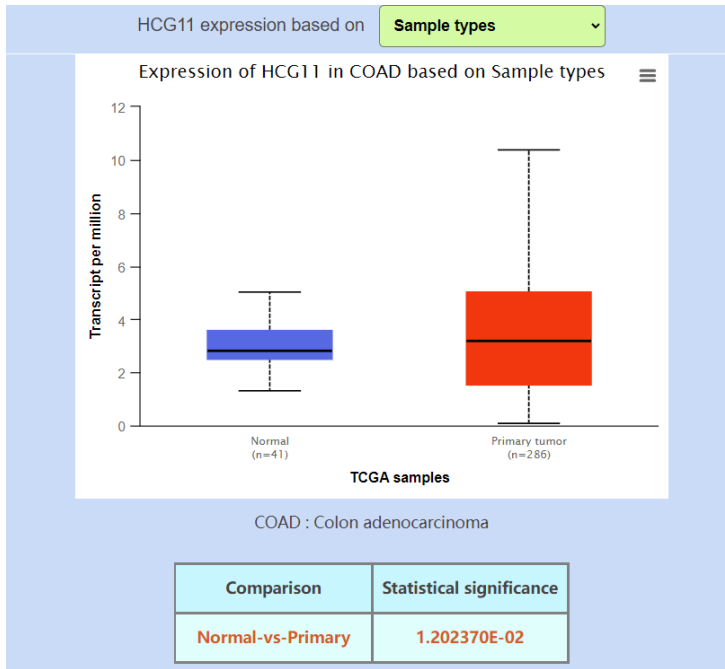

B

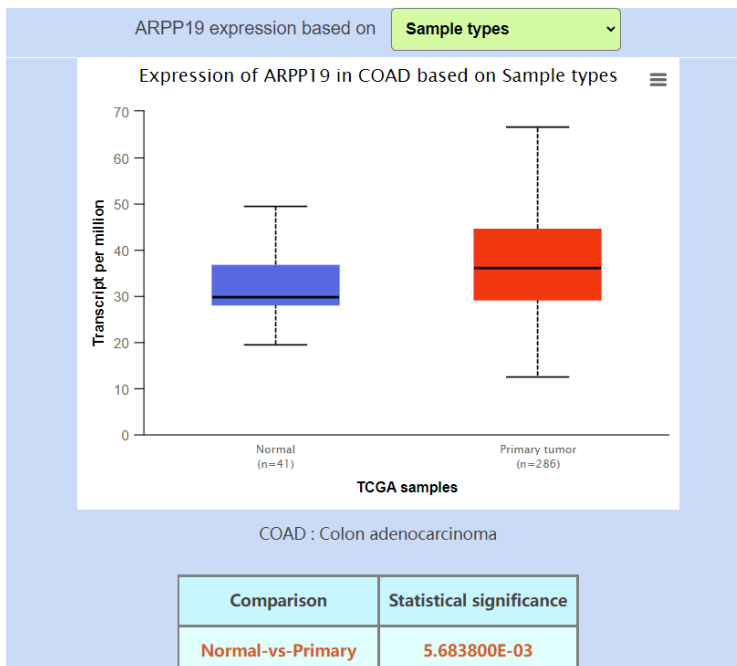

Supplement: Supplementary Materials — Figure S1: (A) HCG11 expression in COAD was predicted on UALCAN. (B) ARPP19 expression in COAD was predicted on UALCAN. [file 9011232.f1.pdf]
